# Polyarticular Sepsis Secondary to Staphylococcus aureus Bacteremia Post-acute Hemodialysis: A Case Report

**DOI:** 10.7759/cureus.30474

**Published:** 2022-10-19

**Authors:** Aparna Peri, Jack Wang, Sanna Salam, Mahmoud Nassar, Erfidia Restrepo

**Affiliations:** 1 Internal Medicine, St. George’s University, New York City, USA; 2 Medicine, Queens Hospital Center, New York City, USA; 3 Internal Medicine, Icahn School of Medicine at Mount Sinai/NYC H&H Queens, New York City, USA; 4 Infectious Disease, Icahn School of Medicine at Mount Sinai, Queens Hospital Center, New York City, USA

**Keywords:** gram-positive bacteremia, staphylococcus aureus bacteremia, hemodialysis related, acute bacterial arthritis, polyarticular septic arthritis

## Abstract

Polyarticular septic arthritis (PASA) is less common than monoarticular septic arthritis (MASA). There is a low incidence of PASA in immunocompetent patients. This case report describes the development of PASA after hemodialysis through the permcath after a single session.

## Introduction

Septic arthritis is an infection of the joints. Bacterial infections are the most common cause; however, a variety of other microorganisms may also be involved. Several risk factors contribute to this condition, including trauma, gout, pseudogout, and rheumatoid arthritis. A hematogenous seeding mechanism is the most common mechanism of infection [1]. *Staphylococcus aureus* is most commonly responsible for hematogenous seeding [1]. There are several symptoms of monoarticular infection, including swelling, pain, warmth, and restricted movement. Depending on the severity of the infection and the patient's age, fever may also be present.

The majority of cases (75%) involve monoarticular joints, while only 15% are polyarticular due to underlying joint disorders such as rheumatoid arthritis or connective tissue disorders. Presented here is a case of polyarticular sepsis that occurred in a patient with acute kidney injury (AKI) on acute dialysis without an underlying etiology for the joint inflammation or a prior immunodeficiency status.

## Case presentation

We report the case of a Venezuelan male in his early 60s who developed AKI as a result of urinary retention status post bilateral nephrostomy tubes placed last month. The patient presented with progressively worsening polyarthralgia, difficulty walking for two days, fever, and diaphoresis for one day. The patient had an uneventful dialysis session the day before his symptoms began. No sick contacts, recent falls, injuries, shortness of breath, nausea, vomiting, rashes, or changes in urine collections have been reported.

In the month prior to admission, the patient had been diagnosed with bladder outlet obstruction, hypernatremia, thrombocytopenia, and anemia. Computerized tomography (CT) abdomen/pelvis revealed bilateral hydronephrosis but no hydroureters. A workup was performed to rule out pyelonephritis. A foley catheter was inserted, and a urinalysis report revealed no evidence of growth. Initially, the patient was treated with gentle hydration, and his blood pressure was monitored regularly. Despite all efforts, the patient’s renal function did not improve. After one week of admission, bilateral percutaneous nephrostomy (PCN) tubes were placed, and a serial basic metabolic panel was monitored. After placing the bilateral PCN tubes, the patient’s blood urea nitrogen level remained elevated at 143 mg/dL, while his creatinine level remained elevated at 13.2 mg/dL, resulting in a decision to begin hemodialysis. The right internal jugular permcath was placed, and the patient began hemodialysis. The patient was hemodynamically stable, with no sign of infection, and a normal complete blood count (CBC) with WBC count, discharged from the hospital and was referred to dialysis and was to follow up with a urology outpatient. Twelve days after the placement of the dialysis catheter, the patient was discharged home. The patient had no prior medical conditions except benign prostatic hyperplasia (BPH), and his family and social backgrounds are unremarkable. Two days after the hospital discharge, he underwent his first outpatient hemodialysis session, which was uneventful, and he returned home relatively well. The outpatient hemodialysis session was conducted two weeks following the placement of the dialysis catheter.

Upon examination, the following vital signs were noted: Temperature: 101.7°F; blood pressure: 98/57 mmHg; pulse: 125 beats per minute; respiratory rate: 20 breaths per minute; and SpO_2_: 89%. The patient was alert and oriented and did not appear to be in any acute distress. There were no notable findings during the physical examination other than diffuse bilateral swelling, redness, and warmth of the shoulders, ankles, knees (right knee was more affected than left), elbows, hands, 3rd metacarpophalangeal joint (MCP), and wrists, as well as red rashes and pain with movement in affected joints. Over the next two days following the prescription of antibiotics, the patient’s symptoms improved. The results of laboratory work are summarized in Table 1.

**Table 1:** Lab results CRP: C-reactive protein; ESR: erythrocyte sedimentation rate; BUN: blood urea nitrogen; eGFR: estimated glomerular filtration rate; CFU: colony-forming unit; MSSA: methicillin-sensitive *Staphylococcus aureus*

	Patient Values	Normal Range
CRP	>300 mg/L	< 5.00 mg/L
ESR	103 mm/hr	0–10 mm/hr
WBC	13.86 * 10^3^/mcL	4.80–10.80^3^/mcL
Creatinine	6.9 mg/dL (on 1st admission) 4.59 mg/dL (on 2nd admission)	0.70–1.20 mg/dL
BUN	192 mg/dL (on 1st admission) 40 mg/dL (on 2nd admission)	6–23 mg/dL
eGFR	13 mL/min/1.72 m^2^	>60 mL/min/1.72 m^2^
Blood cultures growth results from aerobic and anaerobic bottles (peripheral line), DTP from 2nd admission	Grew *Staphylococcus aureus* clusters (MSSA)	
Right knee joint effusion tap and drainage	50 mL of opaque golden fluid, which grew a few positive cocci per oil field	
Urine cultures (from 2nd admission)	Grew >100,000 CFU/mL *Pseudomonas aeruginosa* (ciprofloxacin and levofloxacin resistant)	
Nephrostomy tubes (from 2nd infection)	Grew *Enterococcus faecalis* in the right tubes	

*S. aureus* bacteremia was likely related to tunneled dialysis catheter (TDC) infection, leading to polyarthralgia, septic joints, and echocardiogram report showed mobile echo density on anterior leaflet of the mitral valve, likely diagnosis of infective endocarditis.

The patient developed symptoms 24 hours after undergoing outpatient hemodialysis. Blood cultures from two peripheral sites were positive. Antibiotics were started on the first day of admission following the collection of peripheral blood cultures. The urine culture was positive for *Pseudomonas aeruginosa*, and the nephrostomy culture was positive for *Enterococcus faecalis*, which is different from the blood culture, which was positive for *S. aureus*. Blood cultures from the catheter tip were not obtained as the antibiotic had already been administered for two days, and there was no evidence of infection at the catheter site.

During treatment, the patient was prescribed nafcillin 2 g every four hours and was then switched to cefazolin (2 g/100 mL) upon discharge. The patient’s right knee joint was washed out. A contaminated TDC was removed from the right side, and a new one was placed on the left. PCN tubes were replaced on both sides.

## Discussion

Septic arthritis is an infection of the joint space that can occur in a native joint or a prosthetic joint. The incidence of septic arthritis is higher in patients suffering from rheumatoid arthritis, gout, and pseudogout. Septic arthritis is caused by a range of organisms, including *E. faecalis*, *S. aureus*, *Kingella kingae*, Streptococci, *Neisseria gonorrhoeae*, *Escherichia coli*, *P. aeruginosa*, *Staphylococcus epidermidis*, and *Haemophilus influenza* [2]. The patient has been diagnosed with polymicrobial infection based on two positive blood cultures from two peripheral sites. The urine culture was positive for *P. aeruginosa*, and the nephrostomy culture was positive for E. faecalis, which is different from the blood culture, which was positive for *S. aureus*.

In the United States, two to three septic arthritis cases occur per 100,000 people yearly [3]. Monoarticular septic arthritis (MASA) is commonly affecting the knee or hip. Retrospective case studies suggest only 15% of all septic arthritis cases are polyarticular septic arthritis (PASA), affecting most commonly the knee, elbow, shoulder, and hip [4]. *S. aureus* is the most common causative organism in nongonococcal native joint septic arthritis cases in Europe and North America [5]. Despite treatment, it is reported that there is a mortality rate of 11% in the case of MASA and 50% in the case of PASA [4]. Most cases of PASA are associated with rheumatology, immunosuppression, malignancy, or chronic comorbidities such as diabetes. Developing PASA in an otherwise immunocompetent adult is extremely rare [4].

We have identified two risk factors that are unique to the disease course of the patient in this report. In the context of our patient, he was initially immunocompetent; however, he likely developed AKI in the setting of BPH as pyelonephritis was ruled out on the first admission. Approximately one month after his diagnosis, he developed systemic *S. aureus* bacteremia following a single outpatient dialysis session using a TDC device. The patient developed symptoms 24 hours after undergoing outpatient hemodialysis. Blood cultures from two peripheral sites were positive. Antibiotics were started on the first day of admission following the collection of peripheral blood cultures. Blood cultures from the catheter tip were not obtained, as recommended by the guideline. This is because the antibiotic had already been administered for two days, and there was no evidence of infection at the catheter site [6].

Patients who are predisposed to PASA usually have underlying risk factors and comorbidities such as a prosthetic implant, joint surgery, interventions (e.g., intra-articular injections), immunocompromised state, age > 80 years old, diabetes mellitus, IV drug use, chronic skin infections [4]. Literature reviewing PASA in immunocompetent patients is limited. Chronic kidney disease (CKD) is another nephrological risk factor. Compared to the general population, dialysis patients have a 30- to 50-fold higher risk of mortality secondary to sepsis, especially if they suffer from CKD, while our patient has AKI rather than CKD [7].

TDC under the skin for several centimeters prior to entering the neck vein is intended to reduce the risk of infection and bacterial colonization. While tunneling is designed to reduce infections, the risk of hospitalization for infections in hemodialysis patients with catheters is still two to three times higher than in patients with arteriovenous fistulas or grafts [8]. Gram-positive bacteria are most commonly responsible for causing infections, with *S. aureus* and coagulase-negative staphylococci being responsible for 40%-80% of cases. There are approximately 20%-40% of all catheter-related bloodstream infections (CRBSIs) caused by Gram-negative organisms, 10%-20% due to polymicrobial infections, and <5% due to fungal infections [9]. The prevalence of CRBSIs has been reported to be 1.1-5.5 episodes per 2.7 years of catheterization [9]. Our patient had been on a catheter for less than one month.

Infections caused by *S. aureus* that present with systemic symptoms are associated with a higher mortality rate of 30% to 50% [9]. Our patient presented with multiple joints infected after acute hemodialysis without any comorbidities or risk factors.

## Conclusions

The possibility of PASA can be further enhanced if the patient has had any procedure involving the introduction of foreign objects into their bodies, even if they are considered sterile. Detecting PASA early will increase the chances of an earlier intervention with antibiotics and surgical drainage to reduce morbidity and mortality.
